# Comparative Study on the Seepage Characteristics of Gas-Containing Briquette and Raw Coal in Complete Stress–Strain Process

**DOI:** 10.3390/ma15186205

**Published:** 2022-09-07

**Authors:** Ke Ding, Lianguo Wang, Zhaolin Li, Jiaxing Guo, Bo Ren, Chongyang Jiang, Shuai Wang

**Affiliations:** 1 State Key Laboratory for Geomechanics and Deep Underground Engineering, China University of Mining and Technology, Xuzhou 221116, China; 2 School of Mines, China University of Mining and Technology, Xuzhou 221116, China; 3 State Key Laboratory of Mining Response and Disaster Prevention and Control in Deep Coal Mines, Anhui University of Science and Technology, Huainan 232001, China

**Keywords:** coal and gas outburst, gas-containing coal, complete stress–strain process, permeability, solid-gas coupling

## Abstract

In this study, triaxial compression and seepage tests were conducted on briquette and raw coal samples using a coal rock mechanics-seepage triaxial test system (TAWD-2000) to obtain the complete stress–strain curves of the two samples under certain conditions. On this basis, the different damage forms of the two coal samples and the effect of their deformation and damage on their permeability were analyzed from the perspective of fine-scale damage mechanics. Moreover, the sensitivity of permeability to external variables and the suddenness of coal and gas outbursts were discussed. The results show that the compressive strength of raw coal is 27.1 MPa and the compressive strength of briquette is 17.3 MPa, the complete stress–strain curves of the two coal samples can be divided into four stages and show a good correspondence to the permeability–axial strain curves. Since briquette and raw coal have different structural properties, they present different damage mechanisms under load, thus showing great diversity in the permeability-axial strain curve, especially in the damage stage. The deformation affects the seepage characteristics of briquette mainly in the latter two stages, while it affects raw coal throughout the test. The four stages of the complete stress–strain seepage test of raw coal can well explain the four stages of coal and gas outburst process, i.e., preparation, initiation, development, and termination. Hence, the law of coal permeability to gas variation can be utilized for the coal and gas outburst prediction and forecast. The research results are valuable for exploring the real law of gas migration in coal seams.

## 1. Introduction

Energy, a main driver of the economy, plays a pivotal role in national economic development [1]. Along with the increasing amount and depth of coal mining, problems such as high ground stress and high gas pressure emerge one after another. In the underground mining process in coal mines, the coal body is deformed and damaged by the mining, accompanied by changes in gas permeability characteristics, which is a major cause of gas dynamic damages such as gas gushing from the working face and coal and gas outbursts [2,3,4,5,6]. Therefore, for an effective reduction of the gas-induced adverse effects in underground coal mining, it is of great theoretical significance and practical value to study the law of gas seepage during coal deformation and understand the coal and gas outburst mechanism.

Many scholars believe that briquette shares a similar variation law with raw coal, despite their significant difference in true relative density and apparent relative density (their porosities differ about 4 times and their pore volumes differ 4–10 times). Besides, briquette is easy and efficient to be processed into coal samples. Therefore, briquette can be used as a research object on the general law of gas-containing coal, and the yielded law is applicable to coal seams [7]. At present, researches have been conducted on both briquette and raw coal. By performing comparative tests on the permeabilities of gas-containing briquette and raw coal, Ge et al. [8] found that using briquette samples in place of raw coal samples in the laboratory can only obtain a rough variation law. Pang et al. [9] studied the deformation of coal after gas adsorption during gas pressure change using a self-developed experimental device and discussed the effect of gas on coal based on the existing studies. They concluded that the adsorbed gas would cause expansion and deformation of coal, which weakens its strength and increase its brittleness, so that it becomes more prone to sudden instability damage. Gan et al. [10] investigated the effect of gas pressure on the coal permeability to gas characteristics of coal rock materials in the complete stress–strain process. The results show that at a constant confining pressure, the complete stress–strain curve corresponds well to the seepage rate-axial strain curve, and raising the gas pressure within a certain range can enhance the permeability of coal. Sun et al. [11] conducted triaxial compression and seepage tests on briquette and raw coal using a self-developed gravitational device and found a good correspondence between their complete stress–strain curves and seepage rate-axial strain curves. Zhang et al. [12] explored the deformation characteristics and compressive strengths of briquette and raw coal under triaxial stress conditions based on the results of gas-containing triaxial tests, and concluded that although the two coal samples are similar in the above two properties, they differ significantly in the permeability characteristics. Katarzyna et al. [13] used Brazilian tests to evaluate the work of disintegration of rock resulting from the stresses produced by gas present in its porous structure. However, these research results did not provide a comprehensive comparative analysis on the permeability characteristics of briquette and raw coal. In fact, it is still necessary to further explore the differences in the permeability characteristics between the two during the complete stress–strain process and determine which one is more in line with the actual situation [14,15,16,17,18,19,20,21,22,23,24,25,26,27].

In this paper, gas-containing complete stress–strain tests were conducted on raw coal and briquette by using a coal rock mechanics-seepage triaxial test system (TAWD-2000) to study the similarity and difference in seepage characteristics of the two coal samples during the deformation process. The research results are expected to provide reference for the experimental study using briquette in place of raw coal and serve as an experimental basis for further exploration on the real law of gas migration and the mechanism of coal and gas outbursts in coal seams.

## 2. Test Process

### 2.1. Test Materials

**Figure 1:**
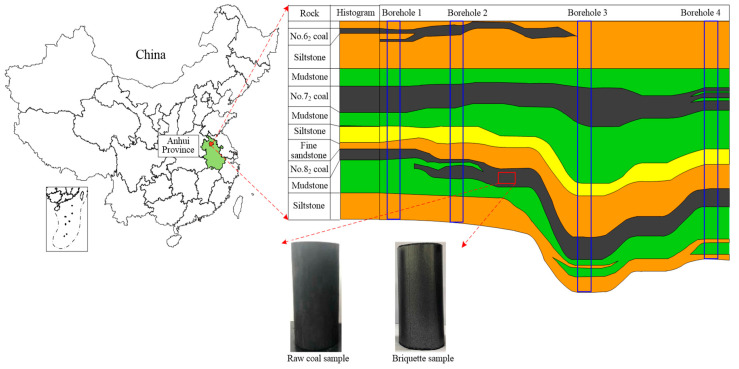
Diagram of the sampling site.

The test coal samples were taken from the No. 8_2_ coal seam of Zouzhuang Coal Mine of Huabei Mining Stock Corporation, China. The average buried depth of the coal seam is 853.7 m, as shown in Figure 1. This coal mine is bounded by the F22 fault and the Dual-Stack fault in the east, the first limestone cropline at the top of the Carboniferous Taiyuan Formation in the south, the Nanping fault in the west, and the #27 exploration line in the north. The No. 8_2_ coal seam is 2.48 m thick on average. The coal body, which belongs to semi-bright coal due to its weak glassy luster, is black, pulverized-fragmented, and has developed endogenous fractures. Soft and fragile, this coal seam is typically prone to coal and gas outbursts.

The preparation of raw coal samples is a difficult task: The raw coal blocks taken from the site were sealed with plastic film and transported directly to the mechanics experiment center of the China University of Mining and Technology where they were cored and processed under the condition that the stratification was perpendicular to the processing axis. According to the test platform and test specifications, the prepared raw coal samples were cylindrical with a height of 95–102 mm, a diameter of about 50 mm, and a parallelism below ±0.05 mm between the upper and lower end surfaces, and they were sealed and stored before the test.

In contrast, the preparation of briquette samples is easier: The raw coal blocks were polished into 40–60 mesh pulverized coal and mixed well with a little water and binder. Then, a certain amount of the mixture was weighed with a balance by experience and put into the mold. After being shaped by a pressure of 200 kN for 20 min, they were made into cylinders with a height of 100 mm, a diameter of 50 mm, and a parallelism of 0.02 mm between the upper and lower end surfaces.

Comparing the two samples, it is clear that the briquette sample had a standard size, a smooth surface and uniform texture, while the raw coal sample had primary damages such as vertical and horizontal fractures, cracks, and holes. Therefore, the raw coal samples with similar density, cracks, and longitudinal wave velocities were selected for the test.

### 2.2. Test Device and Principle

**Figure 2:**
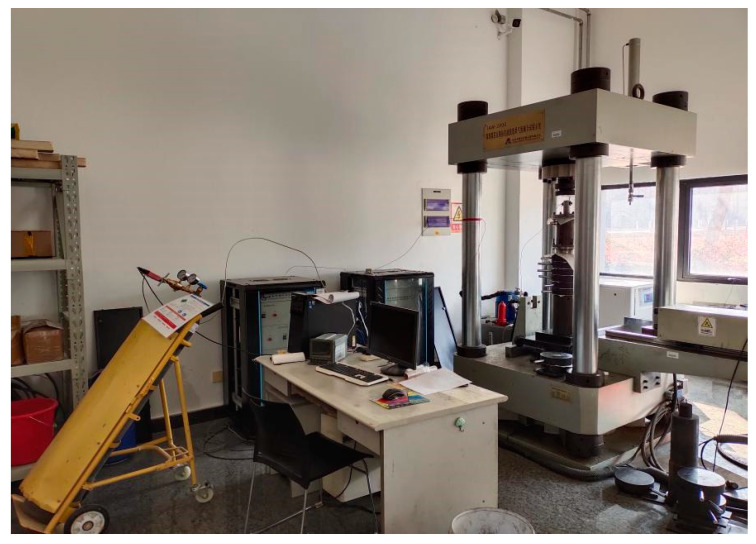
TAWD-2000 coal rock mechanics-seepage triaxial test system.

The coal rock mechanics-seepage triaxial test system (TAWD-2000) of the China University of Mining and Technology was used for the test (Figure 2). The system, which mainly consists of a pressure host system, a pressure and temperature control system, and a microcomputer operating system, can determine the permeabilities of coal rock under different pressure conditions. The maximum working pressures of confining pressure, injection pressure, and axial pressure are 70 MPa, 70 MPa, and 800 MPa, respectively, with the pressures fluctuating within 0.5% in 48 h. The test was conducted at a constant temperature of 25 °C with CH_4_ as the seepage medium.

**Figure 3:**
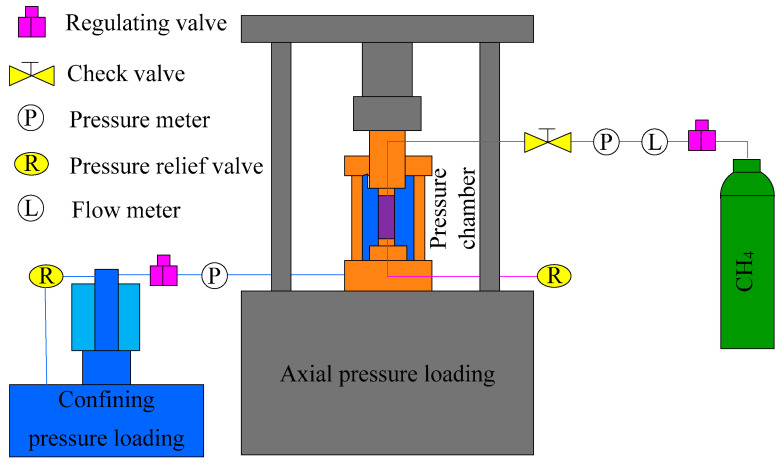
Schematic diagram of the principle of the permeability determination test.

Coal is a porous medium in which the seepage characteristics of gas depend on the number, size, and connectivity of pores and the pressure at both ends of pores in the flow direction. Hence, the permeability characteristics of the sample can be reflected by the relation curve between permeability and other related physical quantities. The principle of the coal permeability measurement test is shown in Figure 3. According to the principle, the steady-state method was adopted in the permeability test: First, different gas pressures were applied to the two ends of a coal sample with a constant pressure difference, so that a certain pressure gradient was formed in the coal sample to promote the flow of gas through the fractures. Meanwhile, the gas flow was measured. When the flow within the coal sample developed into a steady-state flow, the amount of gas flowing through the sample over a while was recorded. The recorded amount can be substituted into the governing Equation (1) to calculate the permeability of the coal sample [19].

(1) $$K=\frac{2p_{0}QL_{\mathrm{coal}}\mu_{{\mathrm{CH}}_{4}}}{A\left(p_{1}^{2}-p_{2}^{2}\right)},$$

where *K* is the permeability, 10^−15^ m^2^; $p_{0}$ is the atmospheric pressure, 0.1 MPa; Q is the gas flow through the coal sample, cm^3^/s; $L_{\mathrm{coal}}$ is the standard length of the coal sample, mm; $\mu_{{\mathrm{CH}}_{4}}$ is the gas dynamic viscosity coefficient, MPa·s; A is the cross sectional area of the coal sample, mm^2^; $p_{1}$ is the inlet pressure, MPa; and $p_{2}$ is the outlet pressure, MPa.

### 2.3. Test Procedure

**Figure 4:**
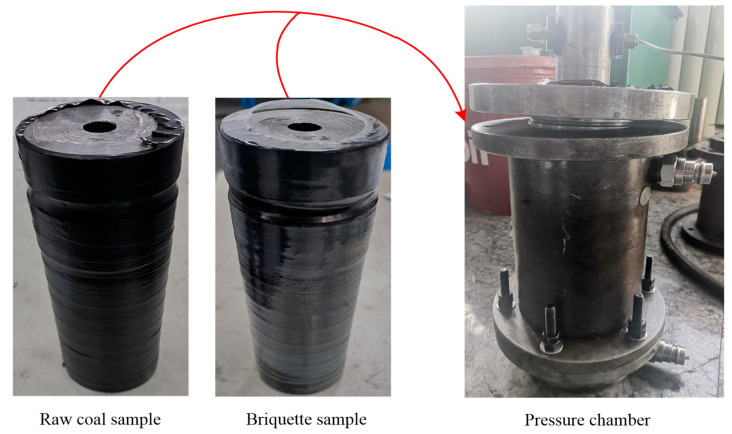
Sealed samples.

In this test, to investigate the changes in gas seepage velocities of briquette and raw coal samples in the complete stress–strain process, a comparative test was carried out according to the geological situation of the No. 8_2_ coal seam in Zouzhuang Coal Mine at a gas pressure of 1.2 MPa and a confining pressure of 4 MPa. The specific procedure and precautions are as follows:

(1) The sample, whose cling film was unwrapped before the test, was fixed with the upper and lower ventilative plates by a thin heat shrinkable film. Then, the fixed sample was wrapped with insulating tape against the hydraulic oil in the pressure chamber during the test. Finally, the sample was wrapped with a thick heat shrinkable film to ensure its air tightness (Figure 4);
(2) Test instruments including the gas pipe, the flow meter, etc., were connected. Axial pressure, confining pressure, and gas pressure were applied to the coal sample in turn, where the amounts of three pressures followed the order: axial pressure of 4 MPa = confining pressure of 4 MPa > gas pressure of 1.2 MPa. Afterwards, the air tightness of the equipment was checked again. After each equipment in the system operated normally, the sample was allowed to fully adsorb gas for 24 h;
(3) The outlet valve was opened to release gas for 30 min until the gas flow stabilized, and then the test started. The loading, controlled by the displacement, proceeded at a rate of 0.002 mm/s until the coal sample finally failed.

## 3. Test Results and Analysis

### 3.1. Comparative Analysis on the Complete Stress–Strain Curves

**Figure 5:**
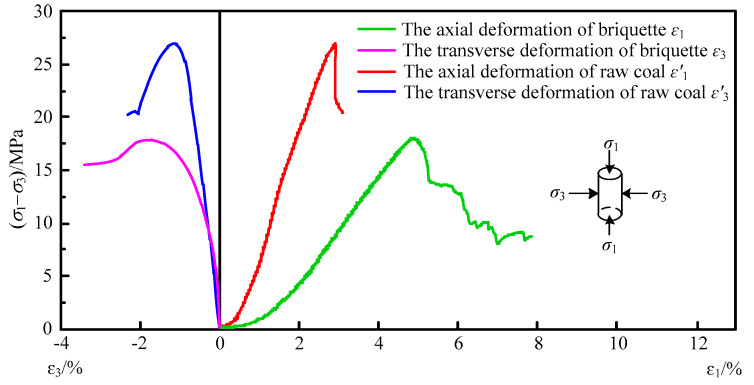
Complete stress–strain curves of briquette and raw coal.

In the triaxial compression test, the complete stress–strain curves of briquette and raw coal show similar variation trends (Figure 5). Both of them can be divided into four development stages: initial compaction stage, elastic deformation stage, plastic deformation stage, and instability damage stage. In the initial compaction stage, the elastic modulus increases with the increase of axial stress and strain, and the stress–strain curve shows a slight upsweep, which results from the compaction of pores and fractures inside the coal sample. In the elastic deformation stage, the stress and strain are linearly correlated with each other, and the elastic modulus becomes constant, following the Hoek–Brown criterion. In the plastic deformation stage, when the axial stress reaches the yield strength, internal damage occurs inside the coal sample, leading to a reduction in the sample’s load-carrying capacity. At this time, the elastic modulus decreases, and the stress–strain curve is no longer linear and curves downward, which is caused by the continuously developing internal damage and new fractures in the sample. In the instability damage stage, after reaching the strength limit, the axial stress begins to decrease with the increase of the strain, which is attributed to the macroscopic cracks penetrating the sample.

The comparative analysis suggests many differences between the test results of the two samples. First, briquette experiences more severe transverse deformation and axial deformation than raw coal, its transverse deformation being twice that of raw coal and its axial deformation being three times that of raw coal. Second, briquette has a lower compressive strength than raw coal, as the compressive strength of raw coal is 27.1 MPa and the compressive strength of briquette is 17.3 MPa, the former being 63.7% of the latter. The elastic modulus of briquette obtained from the test is 0.517 GPa, the elastic modulus of raw coal is 2.306 GPa, the Poisson’s ratio of briquette is 0.22, and the Poisson’s ratio of raw coal is 0.16. Third, the two coal samples exhibit obvious differences in the post-peak deformation and damage stage. Specifically, after the peak, briquette presents a strain softening phenomenon and a gentle decrease in axial pressure, while raw coal shows a stress drop and significant and sharp change in axial pressure, just like the instantaneously and rapidly occurring coal and gas outburst on-site.

The factor that most directly determines coal permeability to gas in coal seams is the development degree of pores and fractures. In the laboratory, both briquette and raw coal have certain primary micropores and microfractures, which are referred to as primary damages [20]. The lower initial permeability of raw coal indicates its much slighter primary damages than briquette. However, as the applied load continues to grow, these primary damages will further develop, extend, and finally connect with each other, leading to a change in permeability of the coal sample.

**Figure 6:**
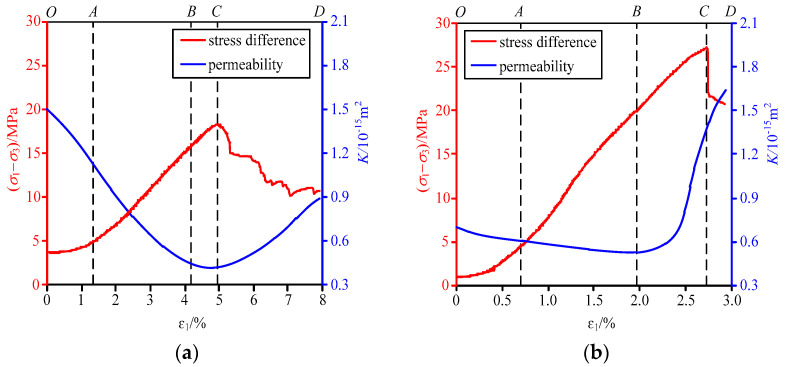
Axial pressure-permeability-strain curves of briquette and raw coal. (**a**) Briquette; (**b**) raw coal.

Based on the changes in strains and permeabilities of briquette and raw coal during the loading stage, the complete stress–strain curves and permeability-strain curves of the two samples were obtained (Figure 6). A correspondence can be found between the complete stress–strain curves and the permeability-strain curves of briquette and raw coal, but the two coal samples have different gas permeability variation laws due to their different damage forms:

(1) Initial compaction stage (OA section): With the rise of axial pressure, the stiffnesses of the two coal samples are enhanced gradually, and the primary fractures are gradually compacted and closed, leading to the shrink of seepage channels. As a result, the permeabilities of both briquette and raw coal decline to some extent;
(2) Elastic deformation stage (AB section): The stress–strain curves of the two coal samples show approximately linear variations. Raw coal is barely damaged internally, so all its primary damages only deform elastically. As its primary micropores and microfractures further close, the coal permeability to gas continues to decline, but such a decline is insignificant owing to its low initial permeability. In contrast, under the action of the external load, the cohesive force of briquette is reduced by the extrusion and dislocation of its particles. Resultantly, the primary fractures between the particles are filled, leading to a rapid decline in its permeability. Besides, its permeability is the most sensitive to stress in this stage;
(3) Plastic deformation stage (BC section): The permeabilities of the two coal samples begin to grow. With the rise of axial pressure, the continuous distributed damages that occurred inside the raw coal create a condition for the stable extension of more and more microfractures, causing plastic deformation. At this time, the permeability of raw coal grows rapidly due to the further development of primary fractures and the formation of new fractures, and the permeability is the most sensitive to stress in the plastic deformation stage. For briquette, the shear movement of its particles facilitates the stable extension of fractures. However, the newly generated fractures are blocked by the detached particles (as they squeeze and displace each other), and thus the permeability grows slowly;
(4) Instability damage stage (CD section): As raw coal experiences a stress drop, where its damage develops from continuous damage to local damage, its fractures with elastic deformation undergo elastic unloading deformation. Consequently, the inelastic strain borne by primary fractures gradually focuses on few fractures generated by the local damage. These large instability-induced fractures enable the gas to pass through smoothly and promote the permeability of raw coal rapidly. However, as briquette only develops based on shear damage, its bearing capacity begins to decline, and its internal structure disenables a sudden stress drop, so its permeability grows only gently.

### 3.2. Relation between Permeability and Axial Stress

**Figure 7:**
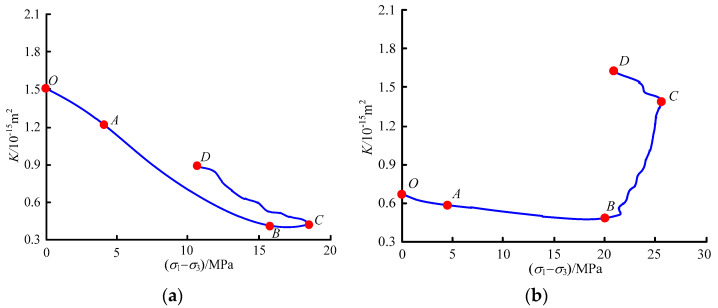
Axial pressure-permeability curves of briquette and raw coal. (**a**) Briquette; (**b**) raw coal.

Based on the data of strains and permeabilities of briquette and raw coal in the loading stage, the permeability-axial stress curves of the two samples were obtained (Figure 7). When the axial stress is below the yield stress, the permeabilities of raw coal and briquette show essentially the same variation trend, that is, they decline with the increase of axial pressure and reach the minimum at the yield stress point. The difference is that the permeability of raw coal diminishes more gently than that of briquette. This shows that briquette is loose and soft, with a large number of voids and a large compressible space; although raw coal has primary fractures, its initial permeability is low and cannot be enhanced obviously by compression.

When the axial pressure exceeds the yield stress, the permeabilities of both coal samples begin to grow, but in obviously different variation trends. In the plastic deformation stage (BC section), briquette exhibits only a smooth rise in permeability, while raw coal shows a steep rise due to the fracture seepage caused by the development of primary fractures and the generation of new fractures.

The peak stress point indicates that the sample reaches its maximum bearing capacity. At this time, the fractures accumulated before the peak reach a critical number, and the sample is on the verge of complete damage, which is a turning point for permeability. The instability damage stage (CD section) reflects the post-peak permeability variation trend. From Figure 7a, it is observed that after the peak stress, raw coal exhibits a rapid stress drop and a steep permeability rise, and this phenomenon indicates that its main fracture emerges and extends suddenly, so the damage is sudden. After raw coal is damaged, its gas pressure gradient soars, thus raising the risk of coal and gas outbursts. From Figure 7b, it can be seen that, unlike raw coal, briquette does not feature suddenness in its parameters, that is, the two samples differ essentially in terms of damage modes and seepage characteristics.

## 4. Discussion

### 4.1. Sensitivity Analysis on Gas Permeabilities of Briquette and Raw Coal

**Figure 8:**
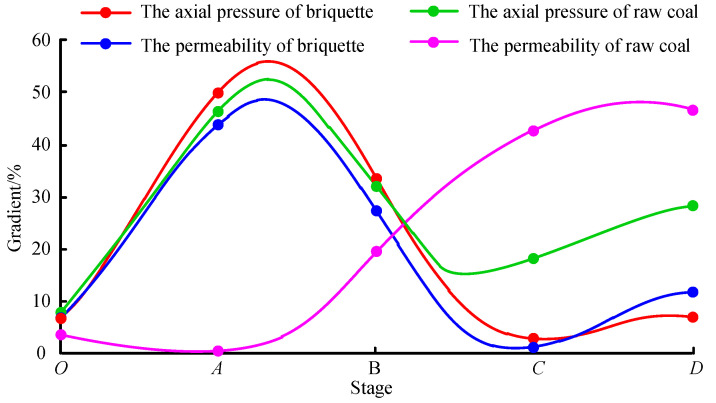
Axial pressure-permeability gradient curves of briquette and raw coal.

In this test, the change in axial pressure affects the permeability of the coal sample at all stages. The entire variation of axial pressure was normalized to analyze the sensitivity of coal permeability to axial pressure in each stage. The axial pressure and permeability exhibit different trends throughout the process and the absolute values of their variations in all stages were summed as 1 for normalization. The axial pressure and permeability gradient curves of the two coal samples are given in Figure 8.

For briquette, its permeability and axial pressure gradients have the same variation trend, both being the largest (55% and 63% respectively) in the elastic deformation stage (AB section) and relatively small in the other stages. This shows that, under triaxial compression, the permeability of briquette is significantly affected by the axial stress and is the most sensitive to axial pressure in the elastic deformation stage.

In contrast, for raw coal, the variation laws of permeability and axial pressure are relatively inconsistent. Nevertheless, in the plastic deformation stage (BC section) and instability damage stage (CD section), the variation laws of the two parameters are roughly consistent, which indicates a relatively high sensitivity of its permeability to axial pressure in these two stages.

### 4.2. Analysis of the Suddenness of Coal and Gas Outbursts

Generally, the occurrence of coal and gas outbursts can be divided into four stages: preparation, initiation, development, and termination. However, some scholars believe that initiation and termination are only two mutation points, while only preparation and development are continuous processes [21]. The seepage tests on briquette and raw coal under triaxial compression can provide new insights into the mechanism of coal and gas outbursts.

Based on the complete stress–strain test and seepage test on raw coal, it is found that the initial compaction stage and elastic deformation stage of the stress–strain-seepage velocity variation belong to the preparation stage of coal and gas outbursts. In this stage, with the rise of axial pressure, the increasing elastic energy from the elastic deformation blocks the gas flow channel, and thus the gas internal energy mounts up obviously. These changes create conditions for the initiation of an outburst. In the plastic deformation stage, plastic deformation occurs because of stable extension of continuously distributed microfractures. At this time, the stress of the sample reaches a limited equilibrium state. In the instability damage stage, the stress drop is a mutation point of outburst initiation. Under the action of axial pressure, the limited equilibrium state of stress is damaged into an instability state. At this time, the sudden release of elastic energy and gas internal energy accumulated in the solid medium of the coal body leads to sudden coal damage, which enables a continuous outburst until the coal body completely loses its bearing capacity. When the subsequent elastic energy and gas internal energy are below the surrounding constraining force, the outburst terminates.

Coal and gas outburst is a kind of dynamic disaster that can be intensely completed within a short time. The difficulty in predicting it lies in its suddenness. As can be concluded from the test, after reaching the peak, the stress of raw coal drops sharply within a short time, accompanied by an increase in gas pressure gradient and a surge of seepage velocity, which is quite close to the occurrence of coal and gas outbursts on site, while the changes in briquette are quite different from the real outburst. Such a result shows that the suddenness of coal and gas outburst is determined by the sudden coal damage under the joint action of in situ stress (axial pressure and confining pressure) and gas pressure, which cannot be accurately reflected by briquette.

## 5. Conclusions

(1) The complete stress–strain curves of briquette and raw coal have similar trends and can be divided into four developing stages: initial compaction stage, elastic deformation stage, plastic deformation stage, and instability damage stage. However, due to the difference between the two samples in structural property, their deformation and damage mechanisms are different. Briquette has a lower compressive strength and experiences a much more severe deformation than raw coal. Therefore, the applicability of briquette in place of raw coal for the simulation study on mechanical behavior needs to be investigated further;
(2) Permeability changes with the deformation and damage of coal, and the variation trend corresponds to the deformation and damage developing stage under load. As the axial pressure rises in the loading process, the permeability of raw coal declines or remains almost unchanged in the initial compaction stage and the elastic deformation stage, and then surges in the plastic deformation stage and the instability damage stage. In contrast, that of briquette plunges in the initial compaction stage and the elastic deformation stage, remains almost unchanged in the plastic deformation stage, and grows steadily in the instability damage stage, but its final permeability is lower than the initial value;
(3) Under triaxial compression, the permeability of briquette is the most sensitive to axial pressure in the elastic deformation stage, while that of raw coal is the most sensitive in the plastic deformation stage. In the test process, compared with the stable damage of briquette, the sudden damage of raw coal is closer to the suddenness of on-site coal and gas outbursts;
(4) The variation law of coal permeability is related to the law of coal deformation, and the four stages of the complete stress–strain-seepage test of raw coal can well explain the four stages of the coal and gas outburst process, i.e., preparation, initiation, development, and termination. Therefore, coal and gas outbursts can be predicted by utilizing the variation laws of coal deformation and damage and permeability in the field.

## Data Availability

The data used to support the findings of this study are included within the article.
